# Provings and Clinical Observations with High Potencies

**Published:** 1893-09

**Authors:** Malcolm Macfarlan

**Affiliations:** Philadelphia, Pa.


### PROVINGS AND CLINICAL OBSERVATIONS WITH HIGH POTENCIES.

MALCOLM MACFARLAN, M. D., PHILADELPHIA, PA.

### TARAXACUM$^{67M}$.

Abdomen greatly distended with gas.

### TUSSILAGO$^{1M}$.

Abdomen distended mostly about the epigastrium; remained so for several days.

### VERBASCUM$^{45M}$.

Cramps about the navel, seems as if the pains were of a twisting kind.

### ANUS.

### ACTÆA-RAC.$^{5C}$.

Costive in a slight degree.

### ADEPS$^{1M}$.

Produced constipation; hard, dry stool; in other provers bowels that had been very costive now move daily; feels weak after stool.

Cramps about the navel, especially before and after stool.

### ALUMINA$^{91M}$.

*Constipation;* stools of a small size, very difficult to expel; dysentery in some provers; can pass urine only during stool. One of the most reliable remedies in the chronic constipation of females.

### ALETRIS$^{45M}$.

Severe pains in rectum or anus; desire to have a stool; the pain in rectum when she had a movement was fearful; felt as if she were forcing a passage, as from an obstruction.

### Adeps$^{1M}$.

Caused the bowels to be loose or open that were constipated; verified this a very great many times. It is my principal remedy in hard, dry, dark stools, difficult of expulsion.

### Apis$^{CM}$.

Cured quickly the most remarkable case of a woman, at the change of life, who, after a natural passage in the morning, would have half a dozen small, straining passages of blood, mucus, and fæces, very offensive. The disease had been of over a year's duration. Verified in several similar cases.

### Aranea$^{45M}$.

Bowels now daily moving, usually four to five days constipated. Urinary symptoms, mentioned elsewhere, are most prominent.

### Arsenic$^{6M}$.

Frequent small stools, of a watery kind.

### Agaric$^{2M}$.

Bowels now move twice a day instead of once.

### Bell.$^{101M}$.

Bowels loose, symptom appearing after several days' proving, primary symptoms.

Bowels constipated very much, dark and hard.

### Capsicum$^{1M}$.

Produced a most violent attack of diarrhœa, coming on suddenly. Verified often.

### Causticum$^{30M}$.

Cured her obstinate constipation; bowels now move daily. The case was one when other curative symptoms of Causticum were indicated.

### CARB-VEG.$^{1M}$.

*Diarrhœa*, with severe griping, lasting several days. A constant and reliable symptom.

### COCHLEAR.$^{10M}$.

Profuse painless diarrhœa, no nausea.

Itching and burning at the anus; twenty movements in his bowels on fourth or fifth day, taking the remedy every hour.

Straining at anus with burning and stinging in several provers.

### COLLINSONIA$^{45M}$.

Cured a most inveterate case of piles with dyspeptic symptoms; frequent good results from this remedy in such cases.

### CUPRUM-ACET.$^{45M}$.

Bowels rather costive, with nausea.

### DIGITALIS$^{CM}$.

Watery diarrhœa, with much thirst; often verified.

### EUONYMUS$^{10M}$.

Bowels move more freely, loose one night four or five times, after proving the remedy for a week, taking it twelve times daily.

### GETTYSBURG SP. WATER$^{45M}$.

Bowels that were regular are now costive; not a prominent symptom.

Bowels loose that were costive. The result of many observations.

### GINSENG$^{46M}$.

Bowels loose, with griping pain.

### HYPERICUM$^{45M}$.

Bowels loose, now move two or three times every day, formerly normal.

Caused symptoms like cholera morbus, lasting three days, afterward became constipated.

### Gelsem.$^{CM}$.

Caused very loose watery stools, after taking remedy for two weeks.

### Lilium-tig.$^{45M}$.

Her bowels which were costive now move more regularly, better than they have ever been, they were always obstinately costive before the proving.

### Merc-corr.$^{5C}$.

After much observation believed to be the most effectual remedy in frequent bloody offensive stools, with great pain in rectum mostly.

### Merc-viv.$^{101M}$.

Had made constipated bowels regular, when stools were small, dark, dry, with severe dyspeptic symptoms.

Highly curative in "bloody flux" or dysentery occurring in the army; violent straining, blood, mucus, pain.

Curative in constipation, with foul breath, severe dyspepsia.

### Merc-prot-iod.$^{2C}$.

Produced movements every few minutes; slight stools, light in color; much mucus. Symptoms noticed on the third day in a child.

### Muriatic-acid$^{5C}$.

Slight diarrhœa and pain in small intestines.

### Mentha-pip.$^{5C}$.

Bowels somewhat loose, griping, wind, frequent eructations.

### Nitric-acid$^{CM}$.

Bowels loose twice daily.

### Nux-vom.$^{94M}$.

Bowels costive when before regular.

### PHYTOLACCA$^{47M}$.

Bowels costive, to a slight degree.

### PLANTAGO-MAJ.$^{5C}$.

Passes much mucus from his bowels, which move oftener than usual.

### PLANTAGO-MIN.$^{11C}$.

Severe pain, with a desire for movement; ineffectual calls to stool.

Bowels constipated and disposition to piles, a frequent condition; disposition to strain at stool.

### POLYGONUM$^{42M}$.

Bowels more regular; relief of severe dyspeptic symptoms; weight at epigastrium; sensation of heat in stomach.

### PSORINUM$^{42M}$.

Bowels move four or five times a day. This symptom lasts for a long time after ceasing the remedy.

The child had always been subject to loose bowels, but since taking the medicine had become costive.

Operate on the woman's bowels too much, move too freely.

Made a wonderful cure of cholera infantum in a scrofulous or delicate child, no vomiting; stools light in color, watery, and offensive.

Bowels which were regular now somewhat constipated.

### PLUMBUM-ACET.$^{1M}$.

Caused very free movements of the bowels six and eight times daily.

Found most useful in constipation, with forms of dyspepsia, with emaciation; with bad or yellow color, exhaustion, foul breath.

### STRONTIA$^{5C}$.

Bowels which moved only twice a week now loose. Frequent cutting pain in lower abdomen and rectum; disposition to strain or bear down; symptoms of dysentery in several provers; no nausea; all the provers had somewhat similar symptoms, after taking the medicine for a week; given hourly.

### SYMPHYTUM$^{15C}$.

Cramp and diarrhœa.

### TEREB.$^{17M}$.

*Piles, bleeding; painful.* One of the most reliable remedies in curing hemorrhoids, especially when bleeding is a prominent symptom.

### THUJA$^{60M}$.

Caused frequent small, painful movements.

### TUSSILAGO$^{1M}$.

Bowels much constipated; distress at epigastrium; complete loss of appetite.

### TEREB.$^{17M}$.

Highly *curative in some forms of piles*, especially attended with bleeding.

$\theta$ useful in *ascarides;* highly curative in itching of the anus.

### TRIF-PRETENS.$^{10M}$.

*Often cured chronic constipation;* bowels often loose after the remedy. Verified this in hundreds of cases since the first proving thirty years ago.

### VERBASCUM$^{45M}$.

Violent diarrhœa on third day; eighteen and twenty movements daily, after the remedy had been taken a week.

### ZINGIBER$^{CM}$.

Loose stools, with much griping; stools which were previously black are now light in color.

### KREOSOTE$^{CM}$.

Caused cutting pain in abdomen; green stools with mucus, nausea, and weakness. Cured (in the greatest heat of summer) when many children were dying of cholera infantum; vomiting, whining, purging, etc. Cured very many bad cases when other medicines were previously tried without any good effect. I earnestly urge all who read this to try this remedy in cholera infantum; it is magical in its curative action generally. Kreosote high was accidentally, in a manner, given as a forlorn hope in a desperate case of cholera infantum, in 1870, with curative effect, and since then often verified.

### URINE.

### ACTEA-RACEMOSA$^{5C}$.

Dark colored urine; passes it frequently and small quantities.

### APIS$^{CM}$.

Wonderfully curative in the difficulty of urination common to children; weakness of the sphincters.

